# The impact of hospital ethical climate on ICU nurses’ ethical behavior: mediated by moral courage

**DOI:** 10.3389/fpsyg.2026.1936074

**Published:** 2026-10-14

**Authors:** Zhiling Zhao, Keke Zhu, Fangqi Zheng, He Wang

**Affiliations:** 1 Coronary Care Unit, Zhengzhou Central Hospital Affiliated to Zhengzhou University, Zhengzhou, Henan, China; 2 Nursing Department, Henan University of Chinese Medicine, Zhengzhou, Henan, China

**Keywords:** ethical behavior, hospital ethical climate, ICU nurses, mediating effect, moral courage

## Abstract

**Objective:**

This study purpose to explore the influence of hospital ethical climate on moral courage and ethical behavior of ICU nurses and to examine the mediating role of moral courage.

**Methods:**

Nurses were selected from the intensive care units (ICUs) of three Grade-A tertiary hospitals in Henan Province by convenience sampling and data collated from General Information Questionnaire, Hospital Ethical Climate Scale, Nurses’ Moral Courage Scale and Ethical Behavior Scale for Nurses-Revised. Pearson correlation analysis was used to evaluate associations among the three variables and structural equation modeling to test the influence of hospital ethical climate and ICU nurses’ moral courage on ethical behavior.

**Results:**

A total of 243 questionnaires were distributed, and 222 valid responses were returned, yielding an effective response rate of 91.4%. Correlation analysis revealed significant positive pairwise correlations among hospital ethical climate, ICU nurses’ moral courage, and ethical behavior (*r* = 0.740 ~ 0.814, *p* < 0.01). Further mediation analysis indicated than there was a positive association between hospital ethical climate and ICU nurses’ ethical behavior (*t* = 17.032, *p* < 0.01), moral courage was also positively associated with ethical behavior (*t* = 3.877, *p* < 0.01). Moreover, moral courage partially mediated the relationship between hospital ethical climate and ethical behavior, with the mediating effect accounting for 32.1% of the total effect.

**Conclusion:**

Moral courage plays a mediating role between perceived hospital ethical climate and ethical behavior in ICU nurses. Accordingly, nursing managers are advised to implement multiple strategies to promote a supportive ethical climate and nurture moral courage, so as to improve ethical conduct among ICU nurses, guarantee patient safety and care quality, and maintain their professional competency and psychological well-being.

## 1 Introduction

With the development of globalization, sociocultural, economic, and political changes, as well as global transformations in information and technology, the importance of integrating nursing practice with ethical principles in healthcare services has become increasingly prominent (Chen et al., 2025). Nurses, as providers of physical, social, cultural, and psychological care, have seen their ethical behaviors in clinical practice become a growing focus of attention and have become an essential component of clinical nursing work standards (Şahin and Alpar, 2024).

The intensive care unit (ICU), characterized by critical illness and high monitoring demands, is a setting for continuous life support and close surveillance (Qiao et al., 2020; He and Huang, 2024). Patients temporarily lose the ability to protect privacy, make decisions, and safeguard their own rights and interests due to impaired consciousness or sedative states (Su et al., 2022). Particularly during the acute phase and clinical fluctuations, their lives are frequently at risk from sudden critical events such as multiple organ failure and septic shock, and urgent situations requiring rapid and accurate decision-making occur frequently (Zheng and Li, 2022; Amina et al., 2025). Consequently, nurses in the ICU frequently encounter ethical challenges. Studies have shown that 67% of ICU nurses experience moral distress (Abbasi et al., 2019), including ethical scenarios such as futile care and false hope, restrictions imposed by treatment costs, and controversies surrounding end-of-life decisions (Emami Zeydi et al., 2022; Zhang et al., 2018; Zhou et al., 2020). Whether nurses can act in accordance with ethical principles in these dilemmas not only directly affects patient safety, dignity, and quality of care (Nazari et al., 2025) but also influences nurses’ own job satisfaction, mental health status, and even their long-term career choices and developmental trajectories (Silverman et al., 2022; Barr, 2020). In this context, how to promote ethical behaviors among ICU nurses and safeguard the rights and interests of critically ill patients has become an important issue in nursing ethics research.

Hospital ethical climate is the ethical perspective of hospital climate (Wang et al., 2018), referring to the shared perceptions and understandings among organizational members regarding policies, procedures, and practices for handling ethical issues, reflecting the organization’s values, norms, and degree of support concerning ethical issues (Olson, 1998). Existing studies have demonstrated that a positive hospital ethical climate is positively correlated with nurses’ ethical decision-making ability, ethical behaviors, and organizational citizenship behaviors (Chen et al., 2025; Xu et al., 2025; Wang S. N. et al., 2022; Wang L. et al., 2022). In the specific clinical environment of the ICU, nurses’ perception of ethical climate is particularly critical—a positive ethical climate is regarded as an important organizational resource that can enhance nurses’ ethical sensitivity and courage to act, provide institutional safeguards and psychological safety for nurses to practice ethical behaviors, and help them cope with moral challenges (Xiang et al., 2026; Jiang et al., 2021; Essex et al., 2023) However, how hospital ethical climate translates into specific ethical behaviors among nurses, and its underlying mechanisms of action, remain to be further elucidated.

Moral courage refers to the willpower and psychological quality that enables individuals to adhere to moral beliefs and take action despite perceiving potential risks such as interpersonal conflicts and public pressure (Ebrahimi et al., 2015; Milliken, 2018). In clinical practice, moral courage is a key psychological resource for nurses to take protective actions when facing ethical dilemmas (Kleemola et al., 2020). Nurses with moral courage are more likely to withstand environmental pressures and fear of punishment, act as advocates for patients’ rights, internalize moral self-discipline as personal beliefs, overcome moral dilemmas, and ensure patient safety (He and Wang, 2021; Yousofvand et al., 2025). Studies have shown that hospital ethical climate can independently explain 57.00% of the variance in ICU nurses’ moral courage (Gao et al., 2023). Furthermore, moral courage partially mediates the relationship between nurses’ moral sensitivity and ethical behaviors (Chen et al., 2025). These findings suggest that moral courage may be a critical psychological variable linking the organizational ethical environment and individual ethical behaviors.

Ethical behaviors refer to actions taken by nurses when facing ethical dilemmas, including the ability to identify ethical issues, make prudent judgments based on professional ethical principles, and ultimately take actions consistent with ethical norms (Chen et al., 2023). Nurses’ ethical behaviors primarily involve behavioral principles such as respect, caring, non-maleficence, and justice (Treviño et al., 2014; Allande-Cussó et al., 2022). In the highly stressful and decision-intensive environment of the ICU, ethical behaviors are specifically manifested in respecting patients’ autonomous choices, maintaining the dignity of dying patients, fairly allocating scarce medical resources, and delivering truthful yet empathetic information to family members in a timely manner (Li et al., 2026). Existing studies have shown that hospital ethical climate is positively associated with nurses’ moral courage and ethical behaviors (Borhani et al., 2021; Hu et al., 2025; Shen et al., 2026), and moral courage has also been confirmed to be closely related to ethical behaviors (Chen et al., 2025). Thus, there may be certain associations among ethical climate, moral courage, and ethical behaviors. However, few studies have currently explored the relationships among the three variables and their interaction mechanisms, leaving management decisions lacking precise theoretical foundations and intervention targets still unclear.

Social Cognitive Theory, proposed by Bandura, emphasizes that individual behavior is determined by the reciprocal interaction of personal factors, environmental factors, and behavior (Bandura, 2001). The theory posits that environmental factors indirectly influence behavior by affecting individuals’ cognitive and emotional personal factors (Xiang et al., 2026). This provides an appropriate theoretical framework for understanding the intrinsic relationships among the above three constructs. In this study, hospital ethical climate corresponds to environmental factors, moral courage corresponds to personal factors, and ethical behaviors correspond to behavior. We aim to explore the relationships among hospital ethical climate, ICU nurses’ moral courage, and ethical behaviors, with a focus on testing the mediating role of moral courage between hospital ethical climate and ethical behaviors, so as to provide theoretical foundations and practical references for promoting and intervening in ICU nurses’ ethical behaviors.

## 2 Methods

### 2.1 Study design and participants

ICU nurses were enrolled from three Grade-A tertiary hospitals in Henan Province by convenience sampling during July 2025. Inclusion criteria were: (1) valid nursing license; (2) > 1 year critical care nursing experience; (3) voluntary participation. Exclusion criteria were: (1) nurses on leave; (2) nurses on training/rotation/internship programs. Minimum sample size was calculated as 10 x number of variables (6 demographic + 12 scale dimensions) (Bentler and Chou, 2016; Ni et al., 2010), augmented by 20% to accommodate potential invalid questionnaires, giving a minimum target sample size of 216 participants.

### 2.2 Instruments

#### 2.2.1 General information questionnaire

The General Information Questionnaire was designed by the current authors to collate basic demographic information of age, gender, educational background, marital status, professional title and other relevant variables.

#### 2.2.2 Hospital ethical climate survey

This scale was developed by Olson (1998), comprehensively covering nurses’ perceptions of ethical climate in various aspects of the work environment, and is the most commonly used instrument for measuring nurses’ perception of hospital ethical climate Koskenvuori et al., 2019. Li et al. (2021) translated and adapted it into a Chinese version, comprising five dimensions (relationships with nurses, patients, physicians, managers, and the hospital) and 25 items. A 5-point Likert scoring method is used: “not at all,” “mostly not,” “uncertain,” “mostly yes” and “completely yes,” giving a total score of 25 to 125, with higher scores indicating a more positive perception of the hospital’s ethical climate. Cronbach’s α coefficient was 0.915, demonstrating good reliability and validity.

#### 2.2.3 Nurses’ moral courage scale

The self-assessment Nurses’ Moral Courage Scale was developed by Numminen et al. (2019), this scale is used to measure clinical nurses’ self-assessed moral courage and has been widely applied in the measurement of nurses’ moral courage levels Wei et al., 2025. Wang et al. (2019) translated, revised, and formed a Chinese version in 2019, which consists of four dimensions (moral integrity, commitment to providing good patient care, compassion and genuine presence with patients, and moral responsibility) with 21 items. A 5-point Likert scale is used: ranging from “completely inconsistent with me” to “completely consistent with me,” scored from 1 to 5 points, giving a total score of 21 to 105, with higher scores indicating greater moral courage. Cronbach’s α coefficient was 0.905 and content validity index (CVI) was 0.910.

#### 2.2.4 Ethical behavior scale for nurses

Developed by Ode (2014), the Ethical Behavior Scale for Nurses (EBSN) was designed to measure nurses’ ethical behaviors in clinical practice. Ode (2019) revised the original scale and developed the Ethical Behavior Scale for Nurses-Revised (EBSN-R), which has been widely used in Japan. Chen et al. (2023) translated and adapted it into a Chinese version through forward translation, back translation, cultural adaptation, cognitive interviewing, pilot survey, and formal survey. The Chinese version comprises three dimensions (good care, risk avoidance and fair care) with 15 items, using a Likert 6-point scoring method ranging from “very inconsistent” to “very consistent,” scored from 1 to 6 points respectively, with total scores ranging from 15 to 90. Higher scores indicate stronger ability of nurses to practice ethical behaviors in clinical settings (Chen et al., 2023). The scale’s dimensional and content design appropriately reflects the concept of ethical behaviors, and its content specifically addresses nurses’ ethical behaviors in daily nursing work, with a moderate number of items and strong feasibility (Chen et al., 2023). The Cronbach’s α coefficient of the scale is 0.892, demonstrating good reliability and validity.

### 2.3 Data collection

This study used the WJX platform, which is widely used for academic research in Mainland China, to distribute the questionnaires. The built-in functions of this platform, including IP address validation and mandatory item settings, were utilized to prevent duplicate responses and missing data from the source, thereby ensuring the technical reliability of the data. The researchers contacted the nursing department heads of the participating hospitals, submitted the ethics approval documents, and clarified the inclusion and exclusion criteria. After obtaining their consent, the electronic questionnaire link was sent to the head nurses of the intensive care units, who then distributed the link with uniform introductory instructions through the departmental work groups. The first page of the questionnaire listed the requirements for completion, the research purpose, and stated that participants were required to provide informed consent before completing the questionnaire. All questions were mandatory with single submission allowed per terminal. A total of 243 questionnaires were collected. Following the exclusion of 21 invalid responses, 222 questionnaires were ultimately included in the statistical analysis.

### 2.4 Data analysis

Questionnaires were validated by two researchers and data uploaded to the database. Statistical analysis was performed using SPSS 27.0 software. All data had a skewness value below 2.58 and a kurtosis value below 1.96 and were considered to show normal distribution, according to the criteria of Hair et al. (2010). Categorical data are presented as frequency and percentage and continuous data as mean ± standard deviation. Relationships among hospital ethical climate, nurses’ moral courage and ethical behavior were evaluated by Pearson correlation analysis. A structural equation model (SEM) to investigate the pathways by which hospital ethical climate and moral courage influenced ethical behavior was constructed using AMOS 29.0. SEM was employed to test the mediation hypothesis based on the following considerations. Compared with traditional regression approaches, SEM simultaneously estimates the measurement and structural models, accounting for measurement error to obtain more accurate parameter estimates. In addition, SEM provides overall model fit indices to comprehensively evaluate the adequacy of the theoretical model in relation to the observed data, making it more appropriate for testing the complex mediation model in this study. Mediation effects were tested by bias-corrected bootstrap confidence interval estimation method with 5,000 resamples. A significant effect was indicated if the 95% confidence interval did not include zero. Model fit was evaluated according to the following criteria: chi-square/degrees of freedom ratio (*χ*^2^/df) < 3.00, root-mean-square error of approximation (RMSEA) < 0.08, goodness of fit index (GFI) > 0.90, normed fit index (NFI) > 0.90, Tucker–Lewis index (TLI) > 0.90, comparative fit index (CFI) > 0.90 and standardized root-mean-square residual (SRMR) < 0.90, Standardized factor loadings > 0.50, Composite reliability (CR) > 0.70, and Average variance extracted (AVE) > 0.5 (Jackson et al., 2009).

### 2.5 Ethical considerations

Ethical approval was granted by the Medical Ethics Committee of Zhengzhou Central Hospital (No. ZXYY2025110). Principles of informed consent, anonymity and non-maleficence were observed. The questionnaire began with a clear informed consent statement and participants could only proceed to the questions after reading this statement and clicking “Start Answering” Complete anonymity was maintained and participants could withdraw from the survey at any stage without giving reasons. Data collected through WJX was stored in a password-protected account accessible only to research team members.

## 3 Results

### 3.1 Social-demographic characteristics

A total of 222 participants were enrolled with mean age of 31.91 (SD = 5.90) years. 86.0% (*n* = 191) were female, 65.8% (*n* = 146) were married, 51.4% (*n* = 114) held intermediate or higher professional titles, 90.5% (*n* = 201) had a bachelor’s degree or higher education level and 70.3% (*n* = 156) had over 5 years of work experience (Table 1).

**Table 1:** The characteristics of the study participants (*N* = 222).

Variable	Categories	Frequency	Percent	Mean	SD
Age				31.91	5.90
Gender					
	Men	31	14.0		
	Women	191	86.0		
Marital status					
	Married	146	65.8		
	Single	75	33.8		
	Others	1	0.5		
Educational background					
	Associate college	21	9.5		
	Bachelor’s degree	196	88.3		
	Master’s degree	5	2.3		
Professional title					
	Junior professional title	108	48.6		
	Intermediate title	103	46.4		
	Senior title	11	5.0		
Years of working					
	1 ~ <2	17	7.7		
	2 ~ <5	49	22.1		
	5 ~ <10	61	27.5		
	10 ~ <20	85	38.3		
	≥20	10	4.5		

### 3.2 Descriptive analysis

The total score for ICU nurses’ perceived hospital ethical climate was 111.10 ± 12.06 with mean score per item of 4.44 ± 0.48. The highest-scoring dimension was relationship with managers (4.53 ± 0.49) and the lowest was relationship with doctors (4.29 ± 0.60). Moral courage produced a total score of 91.20 ± 11.10 and mean item score of 4.34 ± 0.53 with dimension of compassion and genuine presence receiving the highest subscore (4.40 ± 0.54). The total ethical behavior score was 75.40 ± 11.16 with mean item score of 5.03 ± 0.74. Fair care was the lowest-performing dimension (4.64 ± 1.03, Table 2).

**Table 2:** Descriptive results for ethical climate, moral resilience, and ethical competence (*N* = 222).

Items	Scores (mean ± SD)	Means (mean ± SD)
Hospital ethical climate	111.10 ± 12.06	4.44 ± 0.48
Relationship with nurses	18.03 ± 1.95	4.51 ± 0.49
Relationship with doctors	21.46 ± 3.02	4.29 ± 0.60
Relationship with patients	17.85 ± 1.97	4.46 ± 0.49
Relationship with the hospital	26.60 ± 3.11	4.43 ± 0.52
Relationship with managers	27.16 ± 2.91	4.53 ± 0.49
Moral courage	91.20 ± 11.10	4.34 ± 0.53
Moral integrity	30.38 ± 3.85	4.34 ± 0.55
Commitment to excellent care	21.43 ± 2.84	4.29 ± 0.57
Compassion and genuine presence	21.99 ± 2.69	4.40 ± 0.54
Moral responsibility	17.40 ± 2.20	4.35 ± 0.55
Ethical behavior	75.40 ± 11.16	5.03 ± 0.74
Good care	26.06 ± 3.78	5.21 ± 0.76
Risk avoidance	26.12 ± 3.72	5.22 ± 0.74
Fair care	23.22 ± 5.16	4.64 ± 1.03

### 3.3 Correlation analysis

Scores for perceived hospital ethical climate, moral courage and ethical behavior were positively correlated by pairwise analysis (*r* = 0.740 ~ 0.814, *p* < 0.01, Table 3).

**Table 3:** Descriptive data and correlation coefficients between the study variables (*N* = 222).

Variable	Hospital ethical climate	Moral courage	Ethical behavior
Hospital ethical climate	1		
Moral courage	0.814*	1	
Ethical behavior	0.758*	0.740*	1

^*^p < 0.01.

### 3.4 Pathway analysis

SEM pathway analysis was performed to evaluate the influence of hospital ethical climate and moral courage on ethical behavior. Hospital ethical climate was the independent variable and moral courage the mediating variable, jointly acting on ethical behavior. Model fit indices were *χ*^2^/df = 2.267; RMSEA = 0.076; GFI = 0.922; NFI = 0.970; CFI = 0.983; TLI = 0.976, indicating relatively good model fit (Figure 1). All standardized factor loadings of the observed variables on their respective latent variables were significant and exceeded 0.50, indicating that the scale had good construct validity (Table 4). Path analysis revealed that the standardized path coefficient from hospital ethical climate to nurses’ moral courage was 0.830 (*t* = 17.032, *p* < 0.01), indicating a significant positive association between hospital ethical climate and ICU nurses’ moral courage. The standardized path coefficient from hospital ethical climate to ICU nurses’ ethical behavior was 0.555 (*t* = 6.752, *p* < 0.01), also indicating a significant positive association between hospital ethical climate and ICU nurses’ ethical behavior. Furthermore, the standardized path coefficient form nurses’ moral courage to ethical behavior was 0.316 (*t* = 3.877, *p* < 0.01), suggesting that moral courage was similarly significantly and positively associated with ethical behavior.

**Figure 1:**
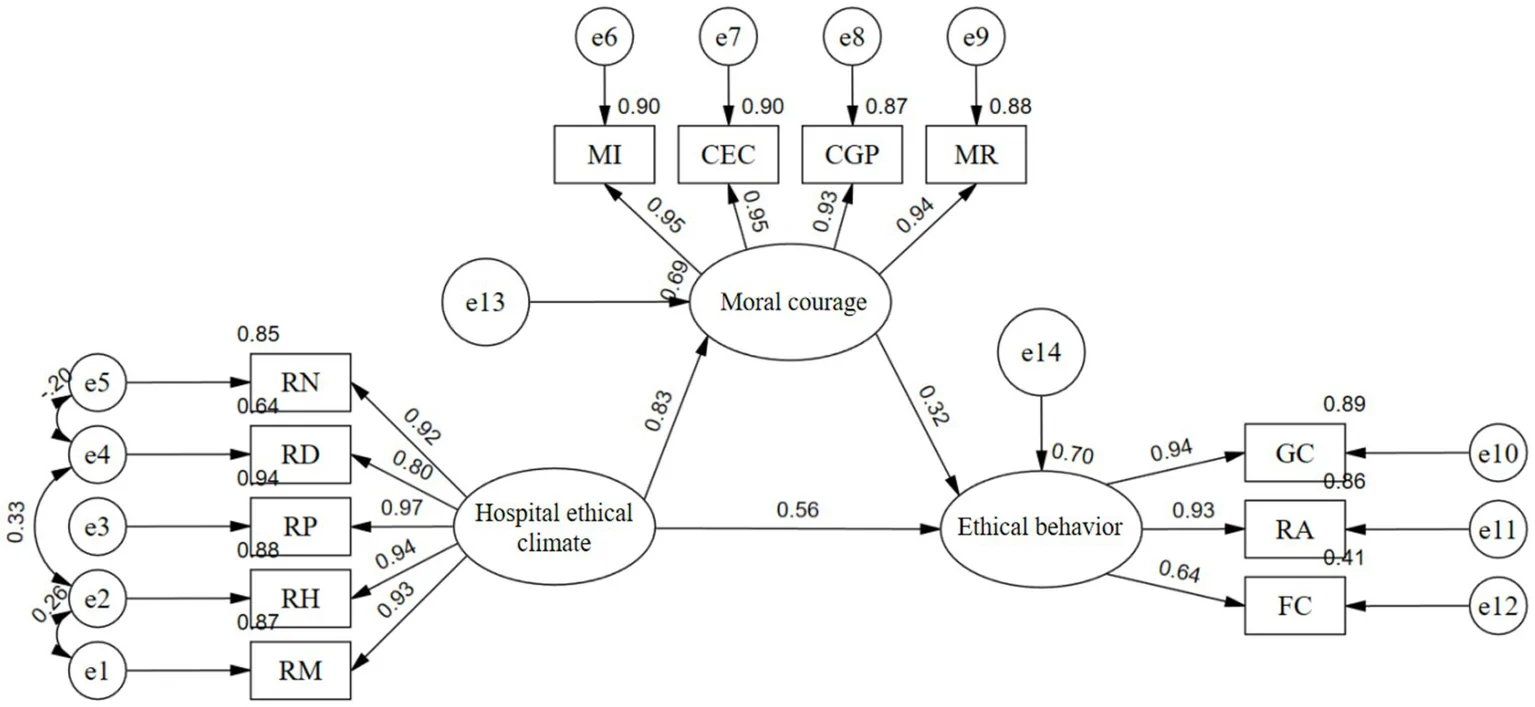
The final model of hospital ethical climate, moral courage and ethical behavior in ICU Nurses. RN, relationship with nurses; RD, relationship with doctors; RP, relationship with patients; RH, relationship with the hospital; RM, relationship with managers; MI, Moral integrity; CEC, commitment to excellent care; CGP, compassion and genuine presence; MR, moral responsibility; GC, good care; RA, risk avoidance; FC, fair care.

**Table 4:** Results of confirmatory factor analysis (*N* = 222).

Construct	Indicator	Std. loading (λ)	CR	AVE
Hospital ethical climate	RM	0.932	0.962	0.837
	RH	0.938		
	RP	0.970		
	RD	0.801		
	RN	0.923		
Moral courage	MI	0.946	0.969	0.887
	CEC	0.949		
	CGP	0.933		
	MR	0.940		
Ethical behavior	GC	0.942	0.881	0.718
	RA	0.928		
	FC	0.637		

The moral courage mediation effect was tested by bias-corrected bootstrap confidence interval estimation, using 5,000 iterations with a 95% confidence interval. Moral courage was mediating variable and ethical behavior dependent variable in a model with hospital ethical climate as independent variable. The total effect value of independent variable on dependent variable was 0.818, direct effect value was 0.555 and indirect effect value was 0.262. The direct effect accounted for 67.9% of the total effect proportion and the indirect effect accounted for 32.1%. The 95% CI did not include zero for either the direct or the indirect effects (Table 5). The moral courage of ICU nurses demonstrated a clear mediation effect between hospital ethical climate and ethical behavior. Hospital ethical climate had a direct effect on ethical behavior and an indirect effect via moral courage, confirming its role as a partial mediation effect.

**Table 5:** Mediation test.

Effects	*β*	SE	95% CI		Effect size
			Lower	Upper	
Total effects					
Hospital ethical climate – ethical behavior	0.818	0.029	0.752	0.868	/
Direct effects					
Hospital ethical climate – ethical behavior	0.555	0.096	0.368	0.744	67.9%
Indirect effects					
Hospital ethical climate – moral courage – ethical behavior	0.262	0.076	0.107	0.409	32.1%

## 4 Discussion

The current study found that high HECS scores were given by ICU nurses (4.44 ± 0.48). Relationships with managers and other nurses were the highest scoring dimensions and relationships with doctors the lowest, consistent with previous studies on nurses in tertiary hospitals in Henan Province (Huang et al., 2023) but differing from the findings of Rivaz et al. (2020). Organizational culture differences and variations in study populations may account for this discrepancy. First, the core of ethical climate construction lies in the organizational micro-environment. ICUs are all high-pressure environments but differences between medical units have a lower impact than differences in organizational management culture (Essex et al., 2023). All current participants came from tertiary hospitals in provincial capitals where there may be greater emphasis on hospital ethical culture. Second, 90.5% participants held bachelor’s degrees or higher, indicating higher education levels. Such nurses had advanced knowledge reserves and skills, greater ethics educational attainment and were predominantly female (86.0%) with higher moral sensitivity (Tuvesson and Lützén, 2016). They were able to identify ethical issues promptly and accurately, handle situational problems professionally and perceived a more positive ethical climate as a result (Ahmed et al., 2024). Finally, supervision by nursing managers coupled with high-frequency interactions and mutual support from fellow nurses established trust and a favorable work atmosphere. Tense nurse-physician relationships may stem from the long-standing “doctor-dominated” Chinese hospital culture. Managers might focus on reconciling nurse-physician relationships, promoting positive collaboration and affirming the professional value of nurses.

The moral courage score (4.34 ± 0.53) was upper-medium for the current cohort of ICU nurses, slightly higher than that found by Pajakoski et al. (2023) or Konings et al. (2022). Cultural background, organizational environment and personal characteristics (Abdollahi et al., 2024; Mollaei et al., 2025; Li H. et al., 2024) may account for these differences. The expression of moral courage is affected by sociocultural context. The collectivist culture in China puts greater emphasis on group cohesion and harmony than the personal autonomy and self-expression favored by the Western individualist culture. Such cultural differences may influence moral courage in the face of ethical dilemma (Mollaei et al., 2025). In addition, it has been shown that the experience of greater moral distress promotes higher moral courage (Li H. et al., 2024). The current ICU nurses would frequently encounter ethical conflicts, such as uncertainty over a patient’s condition, complex family issues and unprofessional behavior by colleagues (Liu et al., 2024). These experiences allow the acquisition of coping skills that enhance moral courage. The dimension of “commitment to good patient care” which measures ethical agency in safeguarding patient rights amid systemic resource constraints and professional practice conflicts, had the lowest mean score. Lower scores indicate insufficient courage to defend patients’ rights in the face of resource shortages, inadequate professional capabilities, compromising behaviors which threaten quality of care or coercive practices.

The ethical behavior was found to be upper-medium (5.03 ± 0.74) for the current cohort of ICU nurses, higher than the findings of Nishimura and Yamada (2022) for Japanese nurses (4.40 ± 0.50) but consistent with previous studies on Chinese oncology nurses (5.07 ± 0.72) (Li Z. Y. et al., 2024). 70.3% of current participants had >5 years of work experience. ICU experience, professional knowledge, nursing and emergency response skills gave participants increased recognition of job value, professional achievement and satisfaction, making them less susceptible to external interference from physicians, patient conditions or time constraints during ethical decision-making (Fang et al., 2025). Ethical behavior was thus practiced more consciously and maturely in the face of complex and high-risk ethical challenges. In addition, the frequency, complexity and urgency of ethical dilemmas and moral decision-making in the ICU demands specialized ethical knowledge and skills (He et al., 2025). Senior experienced nurses are often also clinical educators, with greater comprehension and internalization of core principles of nursing ethics. The “fair care” dimension attracted the lowest scores, possibly due to excessive workloads, human resource shortages and unfavorable resource allocation (Wang S. N. et al., 2022) which prevented ICU nurses from fulfilling moral responsibilities toward patients. Overall, there remains room for improvement in ethical behavior among ICU nurses. Nursing managers might implement interventions to enhance ethical practice while developing unit-specific measures to improve staffing shortages and resource allocation, promoting ethical practice.

Our study found that hospital ethical climate was significantly positively correlated with ICU nurses’ ethical behavior (*r* = 0.758, *P* < 0.01), indicating that the more positive the hospital ethical climate perceived by ICU nurses, the higher their ethical behavior, which is consistent with previous findings (Borhani et al., 2021; Li Z. Y. et al., 2024). Nurses’ perception of hospital ethical climate serves as the most important reference factor when they face clinical ethical issues, and also significantly influences their professional values and nursing service behaviors (Hao et al., 2019). Due to the unique characteristics of ICU patients, ICU nurses frequently encounter ethical issues such as violations of patient autonomy and uncertainty regarding patient treatment, and often engage in more independent thinking about ethical phenomena in their work, making them more sensitive to their perception of hospital ethical climate, thereby leading to higher levels of ethical behavior. Meanwhile, ICU nurses hold positive perceptions of hospital ethical climate, particularly with regard to support from nursing managers and colleagues (Ventovaara et al., 2021). Fostering a positive ethical climate is conducive to promoting nurses’ ethical behaviors so as to provide safe and effective care for patients. When nurses observe that nursing managers and colleagues demonstrate attitudes and behaviors characterized by care and respect toward patients, they themselves learn from and implement these practices. Therefore, hospitals should focus on creating a positive ethical climate, organizing various group activities, cultivating good cooperation among colleagues, and forming cohesive teams. Hospitals should also strive to foster a positive ethical culture, integrate ethical concepts into nurses’ work, and promote nurses’ practice of nursing ethical behaviors through formulating relevant policies and disseminating ethical principles.

The results of this study showed that ICU nurses’ moral courage was significantly positively correlated with ethical behavior (*r* = 0.740, *P* < 0.01), indicating that higher levels of moral courage among ICU nurses were associated with higher levels of ethical behavior, which is consistent with the findings of Jiang et al. (2026). Moral courage is a personal trait rooted in ethical principles and related to ethical concepts, and plays a critical role in ethical practice (Chen et al., 2025). It can prompt nurses to transform ethical evaluation standards into conscious internal compliance with behavior. When faced with ethical issues, nurses with moral courage are more confident, more capable of following inner ethical and moral principles, and more willing to treat patients with honesty, maintain confidentiality for patients, and engage in moral advocacy, thereby helping to overcome moral dilemmas, make correct ethical decisions, and avoid work errors (Mohammadi et al., 2022). A previous study also indirectly demonstrated that nurses with moral courage are better at upholding fairness and justice within organizations and are more inclined to adhere to their own ethical behaviors (Yılmaz and Özbek Güven, 2024). In ICU nursing, patients may be unable to express their needs or protect their rights, making moral courage particularly important for ensuring safe and ethical practice (Asadi et al., 2021). Therefore, nursing managers should place emphasis on cultivating ICU nurses’ moral courage by means such as psychological empowerment and role modeling, so as to enhance nurses’ moral courage levels, thereby helping them comply with ethical principles and norms, protect patients’ rights, and practice ethical behaviors.

Finally, the model results showed that hospital ethical climate had a positive impact on ICU nurses’ ethical behavior, and nurses’ moral courage played a partial mediating role between the two, with an effect value of 0.262, accounting for 32.1% of the total effect. This indicates that hospital ethical climate not only directly influences ICU nurses’ ethical behavior but also indirectly affects their behavioral choices by shaping individual characteristics such as moral courage. Social Cognitive Theory posits that individual behaviors do not exist in isolation but are products of the continuous interaction between intrinsic traits and the surrounding environment (Bandura, 2001). In this study, hospital ethical climate, as an organizational environmental variable, provides nurses with external references for judging correct behavior by conveying clear ethical norms and action guidelines. This environmental signal, once perceived and internalized by nurses, is transformed into their intrinsic moral courage (Mollaei et al., 2025; Pajakoski et al., 2021). Moral courage, as an individual cognitive factor, further drives the occurrence of ethical behaviors—nurses with moral courage, based on their respect for patients’ rights and their strong commitment to nursing professional responsibilities, are able to resolutely choose actions consistent with ethical principles even when facing pressure from superiors or uncertain consequences (Yu et al., 2024). Conversely, if nurses lack moral courage, they may avoid the ethical actions they should take due to fear of conflict or negative evaluation, even in a favorable ethical climate. At the same time, Social Cognitive Theory emphasizes that behavioral outcomes also reciprocally influence the environment and individual cognition—that is, if ethical behaviors taken by nurses out of moral courage receive organizational recognition and positive feedback, this will further reinforce their positive perception of ethical climate and enhance their level of moral courage, forming a virtuous cycle. Therefore, moral courage serves as a critical bridge that translates organizational ethical support into individual actual behavior. In the future, nursing managers should strive to cultivate nurses’ moral courage to help them effectively internalize external ethical guidance into stable and self-conscious ethical practice.

## 5 Limitation

This study employed mediation analysis to examine the mediating role of moral courage in the relationship between perceived hospital ethical climate and ethical behavior among ICU nurses—a group operating in a context of high ethical dilemmas. Despite its contributions, several limitations warrant consideration. First, the cross-sectional design reflects associations only at a single time point, thereby precluding any causal interpretations; longitudinal designs are needed to establish temporal precedence. Second, the exclusive reliance on self-reported measures may introduce common method and social desirability biases, particularly given the sensitive nature of the constructs examined, and future research could benefit from integrating qualitative methods to triangulate findings. Third, the use of convenience sampling restricted to one province in China limits the generalizability of our results, underscoring the need for cross-regional, multi-center replication studies. Fourth, although the sample size met the conventional rule of thumb, this criterion is increasingly viewed as insufficiently rigorous for structural equation modeling (SEM), potentially affecting statistical power. Finally, moral courage accounted for only a portion of the mediating pathway; future investigations should consider additional mediators or moderators (e.g., moral identity, ethical sensitivity) to more fully elucidate the complex mechanisms underlying the ethical climate–behavior relationship, which would in turn inform the development of targeted interventions.

## 6 Conclusion

This study confirmed through path analysis that moral courage plays a partial mediating role between ICU nurses’ perceived hospital ethical climate and their ethical behavior, indicating that a positive ethical climate is not only directly and positively associated with nurses’ ethical behavior but can also be indirectly associated with it through the stimulation of moral courage. This finding provides empirical support for the application of Social Cognitive Theory in the field of nursing ethics, revealing the process mechanism through which environmental factors (ethical climate) are related to behavior (ethical behavior) via individual cognitive factors (moral courage). From a management practice perspective, the core implication of this study is that, in the high-pressure ICU setting characterized by frequent ethical conflicts, hospital administrators should shift their approach to ethical development from one of “institutional constraint” to one of “enablement and activation.” On the one hand, efforts should be made to foster a supportive ethical climate in which nurses perceive organizational respect and tolerance for their moral judgments; on the other hand, moral courage should be incorporated as cultivable psychological capital into nurse training and evaluation systems, using strategies such as scenario simulations and ethical case discussions to strengthen nurses’ ability to translate moral judgment into action in ethical dilemmas. Given the inherent limitations of cross-sectional designs in establishing causal inference, future research should adopt longitudinal designs to further examine the dynamic causal relationships among variables and incorporate a broader range of contextual variables to deepen the understanding of the mechanisms underlying ethical behavior formation.

## Data Availability

The raw data supporting the conclusions of this article will be made available by the authors, without undue reservation.
