# Supplementary material for: The impact of hospital ethical climate on ICU nurses’ ethical behavior: mediated by moral courage
Source: Front Psychol. 2026 Oct 14;17:1936074. doi: 10.3389/fpsyg.2026.1936074 (PMC13713357; doi:10.3389/fpsyg.2026.1936074)
Supplement: Supplementary file 1 [file Data_Sheet_1.PDF]

Questionnaire on the Current Status and Influencing Factors of Ethical Behavior among ICU Nurses

Dear Colleagues,

Hello! My name is Zhao Zhiling, Head Nurse at Zhengzhou Central Hospital. We sincerely invite you to participate in a research project entitled "Current Status and Influencing Factors of Ethical Behavior among ICU Nurses." This study aims to understand the current ethical practice situations, challenges encountered, and influencing factors faced by ICU nurses in their daily work, with the goal of providing scientific evidence for further improving the level of nursing ethical practice, enhancing relevant support policies, and strengthening education and training.

You will be asked to complete an anonymous questionnaire. The questionnaire primarily covers your ethical behavior performance in actual work. Your participation is entirely voluntary. Even if you agree to participate, you may withdraw at any stage of completing the questionnaire without providing any reason, and this will not adversely affect your work, evaluation, or any of your rights. This study involves reflection on ethical practice, and completing the questionnaire may trigger memories of past experiences and cause mild emotional fluctuations. If you feel uncomfortable, you may refuse to answer any questions. The questionnaire is completely anonymous, and no information that could directly or indirectly identify you—such as your name, employee ID, ID number, or phone number—will be collected. All collected questionnaire data will be stored in a password-protected online database, accessible only to the principal investigator. The research findings will be presented in summary form (e.g., overall percentages, means, trend analyses) in research reports or academic papers, and will never include any information that could identify individuals.

Participation in this study may not bring direct personal benefits to you. However, the information you provide will be of great value in gaining an in-depth understanding of the current status of nurses' ethical practice, identifying key influencing factors, promoting ethical development in the nursing profession, and improving the working environment for nurses. You will indirectly contribute to enhancing the quality of nursing services, safeguarding patients' rights, and advancing the nursing profession. If you give your informed consent, please click "Start" to complete the questionnaire. Please respond honestly based on your actual feelings—there are no right or wrong answers. Completing the questionnaire may take approximately 8 - 10 minutes of your time. We sincerely thank you for your participation.

➢ General Information Questionnaire

1.Age ______

2.Gender

☐Men

☐Women

3.Marital status

☐Married

☐Single

☐Others

4.Educational background

☐Associate college
☐Bachelor’s degree
☐Master's degree

5.Professional title
☐Junior professional title
☐Intermediate title
☐Senior title

6.Years of working
☐1～＜2
☐2～＜5
☐5～＜10
☐10～＜20
☐≥20

- Hospital Ethical Climate Survey

	Strongly Disagree	Disagree	Neutral	Agree	Strongly Agree
1.Other nurses will listen to my concerns regarding patient care.					
2.Patients know what nursing services they can expect to receive from nurses.					
3.When I cannot correctly judge a patient's condition, my head nurse or senior colleagues will help me.					
4.Hospital policies can help me resolve difficult patient care problems.					
5.Doctors and nurses trust each other.					
6.Nurses can obtain the necessary information from the hospital to resolve patient care problems.					
7.In the process of care decision-making, my supervisor will support my decision if it benefits the patient.					
8.Nurses reach a consensus on the hospital's philosophy.					
9.Other nurses will help me when I encounter complex patient care problems.					
10.Nurses are willing to apply the necessary information to resolve patient care problems.					
11.My supervisor will listen to my concerns regarding patient care.					
12.When the hospital selects a care plan, the opinions and values of all parties regarding patient care are taken into consideration.					
13.I can participate in the formulation of treatment decisions related to patients.					
14.I trust my supervisor.					
15.When conflicts arise in clinical work, the hospital will address them openly and honestly rather than avoiding them.					

16.Even when there is disagreement about what is best for the patient, nurses and doctors still respect each other's opinions.					
17.I work with a group of competent nurses.					
18.We respect patients' individual wishes.					
19.When my colleagues are uncertain about what is right or wrong in special care situations, I observe that my supervisor will help them.					
20.Regarding patient care issues, the hospital has an awareness of questioning, learning, and seeking creative solutions.					
21.Doctors and nurses respect each other.					
22.In our ward, the care and services that patients receive from other nurses are safe.					
23.My supervisor is a person worthy of my respect.					
24.When I am on duty, I will carry out necessary nursing care when I believe it should be implemented.					
25.In our hospital, nurses are respected by doctors.					

- Nurses' Moral Courage Scale

	Strongly Disagree	Disagree	Neutral	Agree	Strongly Agree
1.Even if I am bullied at work, I will adhere to professional ethics.					
2.Even if I have to confront my own inner fears, I will discuss his/her fears with my patient.					
3.If others insist that I should compromise on adhering to professional ethical principles (patients' dignity, autonomy, justice, and reasonable care), I will raise the discussion that patients have the right to quality care.					
4.When others engage in unethical behavior, I will raise it for discussion, even if it results in negative evaluations of me in my workplace.					
5.If I identify obvious deficiencies in others' professional competence, I will raise it for discussion.					
6.Even though ethical issues often have no definitive answers, I will still participate in the ethical decision-making process of the care team.					
7.Even if others oppose what I believe to be the right answer, I will still participate in the ethical decision-making process of the care team.					
8.If resources required to ensure quality care are insufficient (e.g., staff shortage), I will raise it for discussion.					
9.Even if others wish to remain silent about ethical issues arising in the care process, I will raise it for discussion.					

10.Regardless of the care situation, I will strive to treat every patient as a person with dignity, even if others oppose my approach.					
11.If others engage in violations (e.g., stealing medication from the ward), I will raise it for discussion.					
12.If others attempt to cover up an obvious care error they have made, I will raise it for discussion.					
13.I will express my honest opinion on ethical dilemmas in the care process (e.g., initiating treatment against the patient's will).					
14.I will even go against common nursing practice conventions to support my patient (e.g., if a certain nursing procedure is insufficient to provide quality care, I will spend extra time to serve the patient).					
15.To ensure my patient receives quality care, I will not avoid difficult care situations.					
16.If others compromise on adhering to professional ethical principles (patients' dignity, autonomy, justice, and reasonable care), I will raise the discussion that patients have the right to quality care.					
17.Even if I have to confront my own inner fears, I will offer genuine emotional support to patients suffering from illness.					
18.Even if I am bullied by others, I will not compromise on my patients' right to quality care.					
19.I will admit my own errors in the care process (e.g., administering the wrong medication to a patient).					
20.Regardless of the care situation, I will strive to establish an authentic nurse-patient relationship, even if it is easier for me to maintain a superficial one.					
21.Even if others insist that I should not adhere to professional ethical principles, I will still do so.					

- Ethical Behavior Scale for Nurses

	Strongly Disagree	Moderately Disagree	Slightly Disagree	Slightly Agree	Moderately Agree	Strongly Agree
1.I treat patients differently at work based on my personal preferences.*						
2.At work, I constantly think about how to provide quality nursing care and put it into practice.						
3.To ensure patient safety, I often anticipate potential adverse nursing events that may occur in my work.						
4.At work, I frequently communicate with the medical team to ensure that patients receive accurate information and better obtain their informed consent.						

5.At work, I always strictly adhere to aseptic procedures.						
6.At work, I change the priority order of patient care based on my personal preferences.*						
7.I apply the minimum level of physical restraints to patients in my work to prevent adverse nursing events.						
8.At work, I often consider various factors that may affect patient safety.						
9.At work, I am unwilling to perform tedious and complex nursing tasks.*						
10.At work, I always give my best effort to provide nursing services.						
11.At work, I tend to treat patients with poor compliance negatively.*						
12.In principle, I always protect patients' privacy in my work.						
13.When patients need to sign informed consent forms at work, I strive to create an atmosphere where they can freely express their wishes.						
14.When caring for patients, I cannot fairly balance the physical and mental conditions of multiple patients simultaneously.*						
15.At work, I actively create communication opportunities to listen to patients' opinions.						
